# Patterns and Mechanisms of Legume Responses to Nitrogen Enrichment: A Global Meta-Analysis

**DOI:** 10.3390/plants13223244

**Published:** 2024-11-19

**Authors:** Juan Tang, Wei Li, Ting Wei, Ruilong Huang, Zhuanfei Zeng

**Affiliations:** School of Soil and Water Conservation, Southwest Forestry University, Kunming 650224, China; tangjuan5552023@163.com (J.T.); tingwei0118@163.com (T.W.); hrl13413153274@163.com (R.H.); zzf2002428@163.com (Z.Z.)

**Keywords:** N enrichment, legumes, plant biomass, N_2_-fixation, meta-analysis, global change

## Abstract

Nitrogen (N), while the most abundant element in the atmosphere, is an essential soil nutrient that limits plant growth. Leguminous plants naturally possess the ability to fix atmospheric nitrogen through symbiotic relationships with rhizobia in their root nodules. However, the widespread use of synthetic N fertilizers in modern agriculture has led to N enrichment in soils, causing complex and profound effects on legumes. Amid ongoing debates about how leguminous plants respond to N enrichment, the present study compiles 2174 data points from 162 peer-reviewed articles to analyze the impacts and underlying mechanisms of N enrichment on legumes. The findings reveal that N enrichment significantly increases total legume biomass by 30.9% and N content in plant tissues by 13.2% globally. However, N enrichment also leads to notable reductions, including a 5.8% decrease in root-to-shoot ratio, a 21.2% decline in nodule number, a 29.3% reduction in nodule weight, and a 27.1% decrease in the percentage of plant N derived from N_2_ fixation (%Ndfa). Legume growth traits and N_2_-fixing capability in response to N enrichment are primarily regulated by climatic factors, such as mean annual temperature (MAT) and mean annual precipitation (MAP), as well as the aridity index (AI) and N fertilizer application rates. Correlation analyses show that plant biomass is positively correlated with MAT, and tissue N content also exhibits a positive correlation with MAT. In contrast, nodule numbers and tissue N content are negatively correlated with N fertilizer application rates, whereas %Ndfa shows a positive correlation with AI and MAP. Under low N addition, the increase in total biomass in response to N enrichment is twice as large as that observed under high N addition. Furthermore, regions at lower elevations with abundant hydrothermal resources are especially favorable for total biomass accumulation, indicating that the responses of legumes to N enrichment are habitat-specific. These results provide scientific evidence for the mechanisms underlying legume responses to N enrichment and offer valuable insights and theoretical references for the conservation and management of legumes in the context of global climate change.

## 1. Introduction

Renowned for their unique nitrogen (N) fixation capabilities, legumes play a vital role in the global N biogeochemical cycle [1]. Through symbiotic relationships with rhizobia [2], legumes can convert atmospheric N into ammonia (NH_3_) under ambient conditions, a process known as symbiotic N fixation (SNF) [3]. This process not only meets the N requirements for legume growth but also improves soil structure and fertility, contributing to the maintenance of biodiversity in natural ecosystems [4]. However, with the advent of artificial N_2_-fixing technology, the growing dependence on synthetic N fertilizers in agricultural practices has resulted in global N enrichment [5,6]. From 1980 to 2010, global inorganic N deposition increased from 86.6 Tg N year^−1^ to 93.6 Tg N year^−1^ [7], with projections indicating an increase to 200 Tg N year^−1^ by 2050 [8]. N deposition has triggered various ecological problems, including soil acidification, nutrient imbalances, and biodiversity loss [9,10], while the extensive use of N fertilizers in agricultural practices has further elevated soil N levels. On the one hand, soil N enrichment increases the availability of N for legumes, enhancing functions related to nutrient supply and carbon (C) cycling and potentially boosting legume biomass [11,12,13,14]. On the other hand, soil N enrichment may inhibit rhizobacterial symbiosis, thereby weakening N_2_ fixation and negatively affecting legume growth [2,15,16]. Some studies have shown that adding N fertilizers can effectively enhance the growth and yield of *chickpeas* [17]. However, other research suggests that N fertilizer has no significant impact on the growth and development of *faba beans* [18]. Some scholars argue that N_2_-fixing legumes do not require external N supplementation for growth [19], while other researchers suggest that adding a small amount of external N can promote the optimal growth of legumes [20,21,22]. Since these findings are affected by various research systems, subjects, and focal points, it is difficult to establish a universally applicable understanding across diverse research settings. The growth and development of legumes are also influenced by a range of environmental factors [23], which further constrain our understanding of the impacts of N enrichment on legumes and the underlying mechanisms [24], as well as our ability to assess risks and make informed decisions. Therefore, it is crucial to integrate existing relevant information to help elucidate the general mechanisms by which legumes respond to N enrichment.

N plays a vital role in promoting plant growth, as it is a key component of proteins, nucleic acids, and phospholipids and is involved in the metabolism of essential substances within the plant. It is also an important component of chlorophyll, directly linked to photosynthesis [25]. Although different leguminous plants respond differently to N enrichment [14,26,27], N supply generally promotes legume growth, enhances photosynthesis, increases leaf area, delays plant senescence, and ultimately increases legume biomass [6,28]. Moreover, N supply has a greater impact on legume above-ground biomass than on below-ground biomass, resulting in a reduced root-to-shoot ratio as soil N availability increases [29,30]. According to the optimal allocation theory, plants tend to allocate a large proportion of biomass to the organs, which limits their growth [31]. This suggests that with an increase in external N availability, plants tend to reduce their nutrient investment in below-ground components to enhance their capacity for utilizing photosynthetic resources, thereby resulting in a lower root-to-shoot ratio [32,33,34]. What sets legumes apart from other plants is their unique capability to establish a symbiotic relationship with N_2_-fixing rhizobia, allowing them to assimilate N [3]. During the SNF process, legumes supply rhizobia with essential C sources. In return, rhizobia supports the growth and development of legumes through SNF [35]. The %Ndfa metric is commonly used to quantify the proportion of N derived from the atmosphere. When legumes predominantly rely on SNF, %Ndfa values are relatively high; conversely, when their reliance on SNF is reduced, %Ndfa values are lower [36,37,38]. However, SNF in legumes is an energy-intensive process [39]. Supplementation with externally available N enhances soil N content, which may lead legumes to adjust their N utilization strategies and reduce their reliance on SNF [40,41,42,43]. Based on this understanding, we propose our first hypothesis: N enrichment promotes biomass increase in legumes while potentially reducing %Ndfa.

Climate influences how plants respond to N enrichment by affecting photosynthesis, respiration, and water use efficiency [44,45,46]. Taking leguminous plants as an example, variations in temperature can significantly impact their growth [47]. For instance, an increase in temperature can boost photosynthetic production in legumes [48,49], accelerate the decomposition of soil organic matter [50], and enhance the availability of soil N, thereby improving legume biomass. Conversely, rising temperatures may reduce the competitive ability of leguminous plants within the community, ultimately leading to a decline in their biomass [51]. *Soybeans*, the most widely cultivated leguminous crop in the world, have shown varying responses to increasing temperatures, with reports indicating that rising temperatures may either increase [52,53], decrease [54], or have no significant impact [55] on *soybean* yields. Despite different views on the effects of temperature on plants, most researchers agree that the impact is generally positive [56,57]. Additionally, global warming is expected to affect the rate of N_2_ fixation, as the optimal temperature for nitrogenase activity is 25 °C [58]. Other studies also support the positive impact of warming on N_2_ fixation rates [59,60]. For instance, Ofosu et al. found that increased temperatures can promote the formation and development of *soybean* root nodules [61], thereby enhancing their N_2_-fixing capacity. Global climate change has also led to significant alterations in precipitation patterns [62], which may further exacerbate impacts on ecosystems. Factors such as soil nutrient availability [63], plant growth and development [64], and the C and N reservoirs in the soil [65] are likely to experience disturbances and changes of varying intensity. Specifically, variations in precipitation can alter soil moisture content, which in turn affects the ability of leguminous plants to absorb N, thereby inhibiting their growth and development [66,67]. On the other hand, moist soil conditions promote the growth and reproduction of N_2_-fixing microorganisms, enhancing the N_2_-fixing capacity of plants [68,69]. Water is a key element for plant photosynthesis [70]. Precipitation provides the moisture necessary for photosynthesis, increasing its efficiency and consequently promoting plant growth and enhancing plant yield [71,72]. Diatta et al. reviewed 193 studies on drought stress in *alfalfa* and found that reduced precipitation led to decreased nutrient and water absorption in *alfalfa*, resulting in reduced biomass and impaired biological N fixation [73]. It is noteworthy that the content of soil C and N pools increases with rising precipitation [74,75], which in turn supports and regulates the growth of leguminous plants. For instance, research by Wang et al. [76] demonstrated that increased precipitation significantly boosted the biomass of *Sophora alopecuroides*. Additionally, reduced rainfall can prompt leguminous plants to adapt their survival strategies. For instance, Gei et al. [77] observed that leguminous plants could maintain higher growth rates and water use efficiency by increasing leaf N content in response to decreased moisture in tropical forest environments, thereby retaining a competitive advantage. Appropriate N supplementation can positively affect the physiological functions of plants (e.g., photosynthesis and transpiration) under drought conditions, which promotes the production of photosynthetic proteins and enhances C assimilation [78,79]. However, prolonged drought can lead to the evaporation of soil moisture, impeding the movement of ions within the soil solution and limiting the ability of leguminous plants to assimilate N [66,80]. Clearly, climatic factors influence the regional differentiation of plant growth and traits [81]. Accordingly, we propose a second hypothesis: in regions with abundant water and favorable thermal conditions, N enrichment promotes an increase in the biomass of leguminous plants. In contrast, in arid or cold regions, N enrichment may not significantly enhance the biomass of these plants.

In this context, we conducted a global meta-analysis to assess the responses of leguminous plants to N enrichment. Specifically, we compiled and established a global dataset of N enrichment experiments encompassing various regions, plants, climate types, ecosystems, and soil pH to investigate the impact of N enrichment on leguminous plants at a global scale. First, we analyzed the effects of N enrichment on the biomass and N_2_-fixing capacity of leguminous plants globally. Next, we examined variations in the total biomass of leguminous plants in response to N enrichment across different ecosystems. Lastly, we elucidated the driving mechanisms behind the effects of N enrichment on leguminous plants through a random forest model and correlation analysis. Our aim is to address two core questions: (1) How does N enrichment affect the biomass and N_2_-fixing capacity of leguminous plants? (2) How do climate and soil regulate the response of leguminous plants to N enrichment? We believe that clarifying the ecological adaptation mechanisms and functional changes of leguminous plants within the context of N enrichment is essential for understanding nutrient stress and SNF theories. This will provide a research foundation and theoretical basis for N enrichment management of leguminous plants in agricultural production under scenarios of global climate change.

## 2. Materials and Methods

### 2.1. Data Collection and Dataset Construction

To explore the question of the effect of N enrichment on leguminous plants, we followed the Preferred Reporting Items for Systematic Reviews and Meta-Analyses (PRISMA) to collect eligible pairs of data whenever possible (Figure S1). This study is based on the China National Knowledge Infrastructure (CNKI) (https://www.cnki.net/), Wanfang Data Knowledge Service Platform (WDKSP) (https://www.wanfangdata.com.cn/), and Web of Science (WOS) (https://www.webofscience.com/) database. The keywords used are as follows: Nitrogen addition OR N addition OR Nitrogen fertilizer OR N fertilizer OR Nitrogen deposition OR N deposition OR Nitrogen enrichment OR N enrichment OR Nitrogen treatment OR N treatment OR Nitrogen supply OR N supply OR Nitrogen stress OR N stress AND legume AND plant biomass AND biological nitrogen fixation OR symbiotic nitrogen fixation AND soil. We have collected peer-reviewed papers published up until March 2024. In order to create a valid database, the literature was screened after the search was completed, and the experimental samples included in this Meta-analysis had to fulfill the following criteria: (1) The treatment plants used for the empirical study must be leguminous; (2) the paper must contain a treatment group (with N added) and a control group (without N added); and (3) for the same experiment, it is ensured that environmental factors such as the experimental site, climatic conditions, and soils are kept the same for the treatment group and the control group. In addition, if the legumes in the literature belonged to the same species but were in different growth periods, their observations were included in the analysis as independent situations. Information presented in tables, text, and attachments in the study will be recorded directly, and data shown in graphical form will be extracted using the GetData Graph Digitizer (2.26).

**Figure 1:**
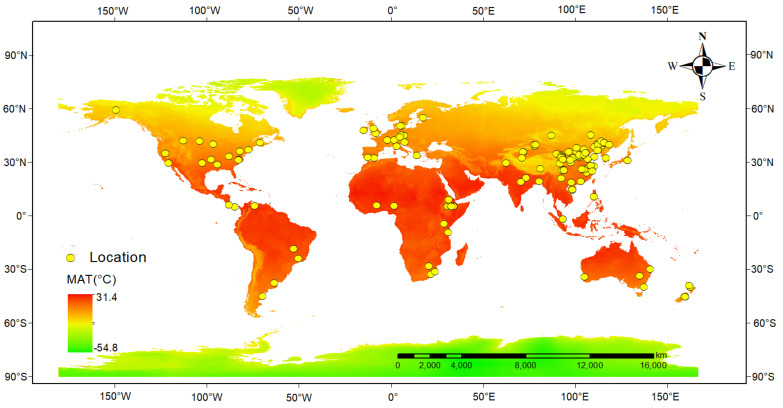
Location distribution of the study area. Base map derived from the World Climate Database; the yellow dots indicate the test sites, and MAT indicates the mean annual temperature.

Additionally, we documented in detail the types of ecosystems at each research site, the plant species, the types and application rates of N fertilizers, the duration of N experiments, and the sites of these experiments. In order to explore the influence of soil properties and the climatic conditions at the research sites on leguminous plants, we also extracted soil characteristics from the experimental plots, including soil total nitrogen (STN), soil organic carbon (SOC), soil pH, site-specific latitude and longitude, altitude, mean annual temperature (MAT), and mean annual precipitation (MAP). If the altitude, MAT, and MAP of the experimental site were not provided in the original literature, they were extracted by WorldClim2 based on the latitude and longitude of the experiment site [82]. The aridity index (AI) was obtained from the Global Aridity Index and Potential Evapotranspiration Database based on the latitude and longitude of the experiment site [83]. If the literature only reports soil organic matter (SOM), SOC can be calculated by dividing SOM by the conversion factor of 1.724. Additionally, we computed the soil C/N ratio using STN and SOC. Furthermore, for instances where different variables in the literature were reported in various units, we standardized the units according to the conversion formulas between them. In total, we constructed a paired dataset of 2174 observations on the impact of N enrichment on leguminous plants derived from 162 peer-reviewed articles. This dataset comprises 810 pairs of total biomass, 675 pairs of root-to-shoot ratios, 717 pairs of plant N content, 142 pairs of plant N uptake, 711 pairs of nodule numbers, 552 pairs of nodule weights, 355 pairs of %Ndfa, and 156 pairs of nitrogenase activity. Specifically, the altitude range was 1–4730 m, the MAP range was 34–2600 mm, the MAT range was −8–28.26 °C, the AI range was 0.02–1.89, the STN range was 0.27–5.4 g/kg, SOC range was 2.26–56.85 g/kg, soil pH range was 3.99–9.2, and the N addition rate range was 7.7–600 kg N ha^−1^. Approximately 69% of leguminous plants were sourced from temperate regions, and 45.6% of the N addition experiments were conducted in cropland ecosystems. In 62.8% of the experiments, Urea was the type of N fertilizer used, while 19.1% utilized mixed N types. About 38.5% of the N application rates were ≤100 kg N ha^−1^, and 82.8% of the N addition experiments were <1 year. Furthermore, 46.7% of the experiments were field studies, while 53.4% of the experiments were conducted indoors, with the geographical distribution of the research locations illustrated in Figure 1. In summary, this dataset covers a diverse range of climatic zones and soil properties.

To compare the total biomass of leguminous plants in response to N enrichment across different experimental regions, methodologies, plant species, and ecosystems, we categorized the database into twelve groups. These groups were organized by absolute latitude (arctic/sub-arctic (>50° N/S), temperate (35–50° N/S), sub-tropical (23.4–35° N/S), tropical (23.4° N–23.4° S)); by plant species (woody and herbaceous); by altitude (≥1000 m and <1000 m); by MAP (≥800 mm and <800 mm); by MAT (<15 °C and ≥15 °C); by AI ((humid (AI > 1), sub-humid (0.65 < AI ≤ 1), dry sub-humid (0.5 < AI ≤ 0.65), semi-arid (0.2 < AI ≤ 0.5), arid (AI ≤ 0.2)); by terrestrial ecosystems (grassland ecosystems and cropland ecosystems); by soil pH ((acid soil (≤6.5), neutral soil (6.5–7.5), alkali soil (>7.5)); by N application type (urea, ammonium N (NH_4_^+^-N), nitrate N (NO_3_^−^-N), and mixed (NH_4_NO_3_ and NO_3_^−^ + NH_4_^+^)); by N application rate (≤100 kg N ha^−1^ and >100 kg N ha^−1^); by duration of the experiment (<1 year and ≥1 year); and finally, by experimental site (field and indoor).

### 2.2. Meta-Analysis

First, for some of the missing values of STN, SOC, and soil pH, we chose the Multiple Imputation by Chained Equations (MICE) with Random Forests to fill in the gaps. The results showed that the observed values were well fitted to the interpolated values and tended to converge (Figure S2), suggesting that this method is suitable for our dataset.

Subsequently, we used a random-effects model weighted natural log response ratio ln⁡(RR) to reflect the effects of N enrichment on legumes. For this purpose, the mean, standard deviation (SD), and sample size (n) of each treatment and control group were extracted, and if the standard error (SE) instead of SD was demonstrated in the literature, the SD was calculated as follows:

(1) SD=SE√n

If SD or SE was not reported in the article, for missing values we used the mean coefficient of variance of the entire dataset for supplementation. If n was not reported, we then supplemented using the median of the sample size of the entire data set [84].

The following is how the ln⁡(RR) was calculated [85]:

(2) $$\ln\left(RR\right)=ln\frac{{\bar{X}}_{t}}{{\bar{X}}_{c}}=\ln\left({\bar{X}}_{t}\right)-\ln\left({\bar{X}}_{c}\right)$$

where ${\bar{X}}_{t}$ and ${\bar{X}}_{C}$ are the mean values of the treatment and control groups, respectively. The weighted mean response ratio $ln({RR}_{+})$ was calculated as follows:

(3) $$\ln\left({RR}_{+}\right)=\frac{\sum_{i=1}^{m}w_{i}^{*}ln(RR_{i})}{\sum_{i=1}^{m}w_{i}^{*}}$$

where m is the number of groups in each corresponding compared group and $w_{i}^{*}$ is the weight factor of the corresponding comparisons, which was computed as follows:

(4) $$w_{i}^{*}=\frac{1}{v_{i}^{*}}$$

where $v_{i}^{*}$ is the study i variance in the group, which was calculated as follows:

(5) $$v_{i}^{*}=T^{2}+V_{i}$$

where $T^{2}$ is the between-study variance and $V_{i}$ is the within-study variance for group i [86], which was computed as follows:

(6) $$v_{i}^{*}=\frac{{SD}_{t}^{2}}{n_{t}{\bar{X}}_{t}^{2}}+\frac{{SD}_{c}^{2}}{n_{c}{\bar{X}}_{c}^{2}}$$

where, ${SD}_{c}$ and ${SD}_{t}$ are the SD of the control group and the treatment group, respectively, and $n_{c}$ and $n_{t}$ are the sample size of the control group and the treatment group, respectively, of group i.

The standard error $s(\ln\left(RR_{+}\right))$ of the weighted average response $ln(RR_{+})$ and the 95%CI was calculated as follows:

(7) $$s(\ln\left(RR_{+}\right))=\sqrt{\frac{1}{\sum_{i=1}^{m}w_{i}^{*}}}$$

(8) $$95\%CI=\ln\left(RR_{+}\right)\pm 1.96s(\ln\left(RR_{+}\right))$$

N enrichment was considered to have a significant effect on the target variables when the 95%CI did not overlap with 0. For a better interpretation, the mean effect size was transformed back to the percentage change induced by the experimental N addition treatment [87], which was computed as follows:

(9) $$Effect\;size\left(\%\right)=(\left(\exp\left(\ln\left(RR_{+}\right)\right)-1\right)100\%$$

This study applied funnel plots and Egger regression to detect publication bias in all studies (Figures S3 and S4). Meta-analysis was performed using the metafor package in R 4.2.3 for overall and subgroup analyses.

### 2.3. Data Analysis

Data are standardized by taking logarithms of the variables in the data set to reduce heterogeneity between the data and sensitivity to differences. To illustrate how N enrichment affects legumes, we used correlation analysis to investigate associations between legume growth traits and N_2_ fixation metrics, along with environmental factors or N addition rate. In the correlation analysis, *p* < 0.05 was considered significant, and the data were visualized using ggplot2.

Additionally, this study also employed the random forest model to analyze the relative importance and significance of the effects of various environmental factors on legumes, which was implemented using the random forest package. In this framework, significance is measured by the value of “percentage of increase in mean square error” (Increase in MSE (%)) in Random Forests, where higher values of MSE% imply more important variables. The significance of each variable is output through the rfPermute package [88]. Data analysis and visualization were conducted using R (4.2.3).

## 3. Results

### 3.1. A Comprehensive Analysis of the Responses of Legume Biomass, N Accumulation, and Symbiotic N_2_ Fixation to N Enrichment

**Figure 2:**
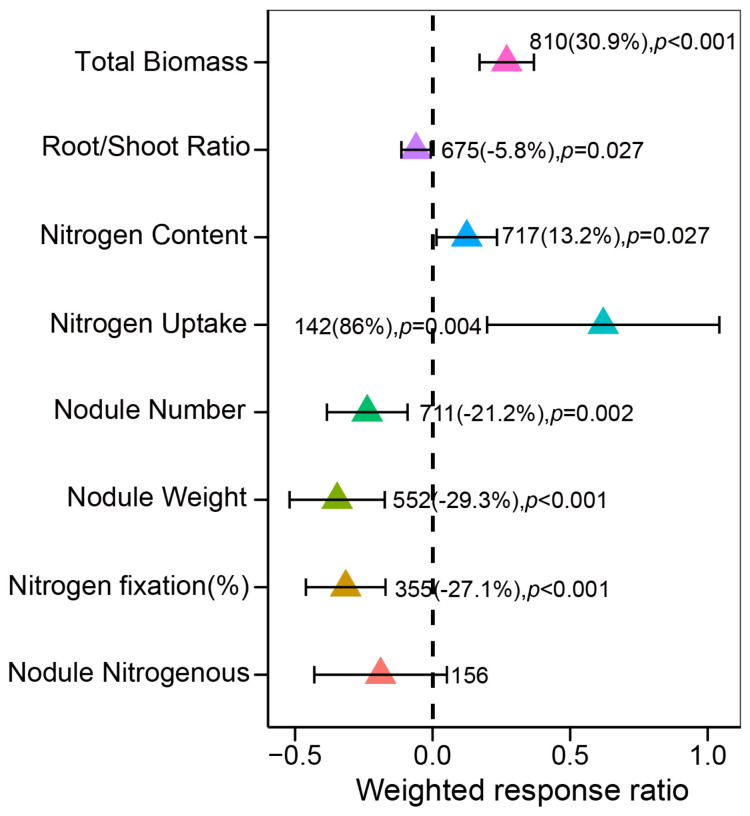
Overall analysis of the impact of nitrogen enrichment on leguminous plants. The black line in the figure represents the 95% confidence interval. Numbers outside this bracket indicate the sample size, numbers inside the bracket indicate the percentage change in the variable, >0 indicates a positive effect, <0 indicates a negative effect, and *p* < 0.05 indicates a significant response.

The global scale N enrichment significantly increased the total biomass of leguminous plants by 30.9% (*p* < 0.001, n = 810). In contrast, under N enrichment, the root-to-shoot ratio of leguminous plants was significantly reduced by 5.8% (*p* = 0.027, n = 675). Notably, the N uptake and tissue N content of leguminous plants under N enrichment increased significantly by 86% (*p* = 0.004, n = 142) and 13.2% (*p* = 0.027, n = 717), respectively. However, N enrichment inhibited both nodule number (−21.2%, *p* = 0.002, n = 711) and nodule weight (−29.3%, *p* < 0.001, n = 552), while %Ndfa decreased by 27.1% (*p* < 0.001, n = 355). Furthermore, the nitrogenase activity of legume nodules (*p* = 0.125, n = 156) did not show a significant response to N enrichment (Figure 2 and Figure S5).

### 3.2. Subgroup Analysis of the Impact of N Enrichment on Total Biomass of Leguminous Plants

**Figure 3:**
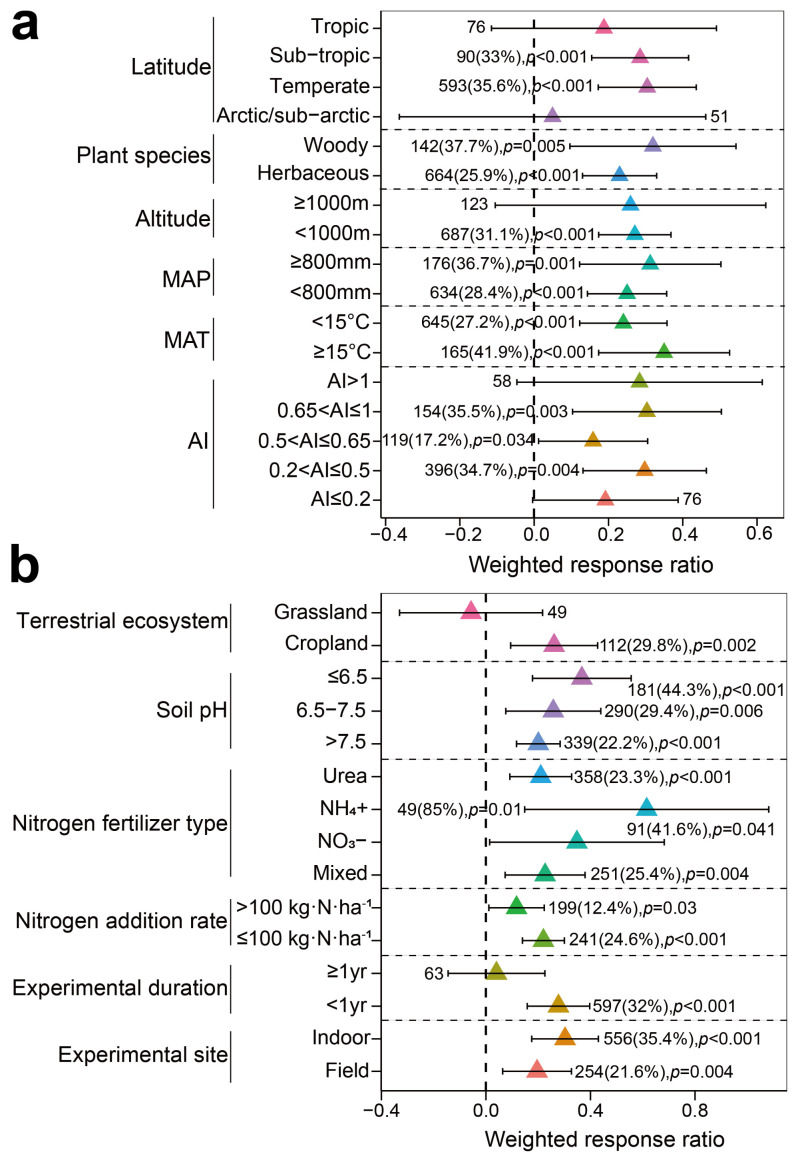
Subgroup analysis of the effect of nitrogen enrichment on the total biomass of leguminous plants. The black line in the figure represents the 95% confidence interval. Numbers outside these parentheses indicate sample size, numbers inside parentheses indicate percentage change in the variable, >0 indicates a positive effect, <0 indicates a negative effect, and *p* < 0.05 indicates a significant response. (**a**) subgroup analyses of Latitude, Plant life form, Altitude, MAP, MAT, and AI in total biomass of leguminous plants; (**b**) subgroup analyses of Terrestrial ecosystem, Soil pH, Nitrogen fertilizer type, Nitrogen addition rate, Experiment duration, and Experiment site in total biomass of leguminous plants.

Both subtropical (33%, *p* < 0.001, n = 90) and temperate regions (35.6%, *p* < 0.001, n = 593) exhibited a significant response in total biomass of leguminous plants to N enrichment. However, in tropical (*p* = 0.224, n = 76) and arctic/sub-arctic regions (*p* = 0.814, n = 51), N enrichment did not show a significant effect on total biomass of legumes (Figure 3a). The response of leguminous plant total biomass to N enrichment varied under different geographical factors. When altitude was <1000 m, the total biomass of legumes significantly increased by 31.1% (*p* < 0.001, n = 687), whereas when the altitude was ≥1000 m, N enrichment had no significant effect on total biomass (*p* = 0.163, n = 123) (Figure 3a). Results from the MAT groupings indicated that when the MAT was ≥15 °C, the total biomass of legumes significantly increased by 41.9% (*p* < 0.001, n = 165), which was considerably more than the total biomass increase in legumes when the MAT was <15 °C (27.2%, *p* < 0.001, n = 645) (Figure 3a). The MAP grouping results showed that when MAP was ≥800 mm and <800 mm, the total biomass of legumes increased significantly by 36.7% (*p* = 0.013, n = 176) and 28.4% (*p* < 0.001, n = 634), respectively (Figure 3a). According to the AI grouping results, when 0.65 < AI ≤ 1 and 0.2 < AI ≤ 0.5, N enrichment resulted in an increase in total biomass of leguminous plants by 35.5% (*p* = 0.003, n = 154) and 34.7% (*p* < 0.001, n = 396), respectively, which was significantly greater than the total biomass increase when 0.5 < AI ≤ 0.65 (17.2%, *p* = 0.034, n = 119). In contrast, when AI > 1 (*p* = 0.092, n = 58) and AI ≤ 0.2 (*p* = 0.055, n = 76), N enrichment had no significant impact on the total biomass of leguminous plants (Figure 3a).

Soil pH also plays a crucial role in the growth of leguminous plants. Grouping results for different pH levels showed that in acidic and neutral soils, N enrichment significantly increased the total biomass of leguminous plants by 44.3% (*p* < 0.001, n = 181) and 29.4% (*p* = 0.006, n = 290), respectively, while in alkaline soils, total biomass only increased by 22.2% (*p* < 0.001, n = 339) (Figure 3b). The significant enhancement of total biomass in woody legumes (37.7%, *p* = 0.005, n = 142) and herbaceous legumes (25.9%, *p* < 0.001, n = 664) due to N enrichment varied across different plant species (Figure 3a). Additionally, in cropland ecosystems, the total biomass of legumes increased markedly by 29.8% (*p* = 0.002, n = 112), whereas in grassland ecosystems, the total biomass of legumes showed no significant response to N enrichment (*p* = 0.684, n = 49) (Figure 3b). Variations in experimental methodologies also influenced the response of total biomass to N enrichment. The results from grouping based on N fertilizer application rates indicated that when the N application rate was ≤100 kg N ha^−1^, N enrichment significantly enhanced the total biomass of legumes (24.6%, *p* < 0.001, n = 241), a greater increase compared to rates >100 kg N ha^−1^ (12.4%, *p* = 0.030, n = 199) (Figure 3b). Grouping by N fertilizer type revealed that different forms of N significantly affected the total biomass of legumes. Urea and mixed N forms increased total biomass by 23.3% (*p* < 0.001, n = 358) and 25.4% (*p* = 0.004, n = 251), respectively. However, these average increments were still lower than those observed with NO_3_^−^-N (41.6%, *p* = 0.041, n = 91) and NH_4_^+^-N (85%, *p* = 0.01, n = 49) (Figure 3b). Regarding the duration and site of experiments, when the experimental duration was <1 year, the total biomass of legumes significantly increased by 32% (*p* < 0.001, n = 597), whereas no significant response was detected for durations ≥1 year (*p* = 0.67, n = 63) (Figure 3b). Indoor and field experiments resulted in substantial increases in the total biomass of legumes of 35.4% (*p* < 0.001, n = 556) and 21.6% (*p* = 0.004, n = 254), respectively (Figure 3b). These findings suggest that the impact of N enrichment on the total biomass of legumes varies by ecosystem. Factors such as regional differences, climatic conditions, soil pH, plant species, ecosystem types, and experimental methods contribute to the variability observed in the total biomass of legumes (Figure S6).

### 3.3. Driving Patterns of Legume Responses to N Enrichment

**Figure 4:**
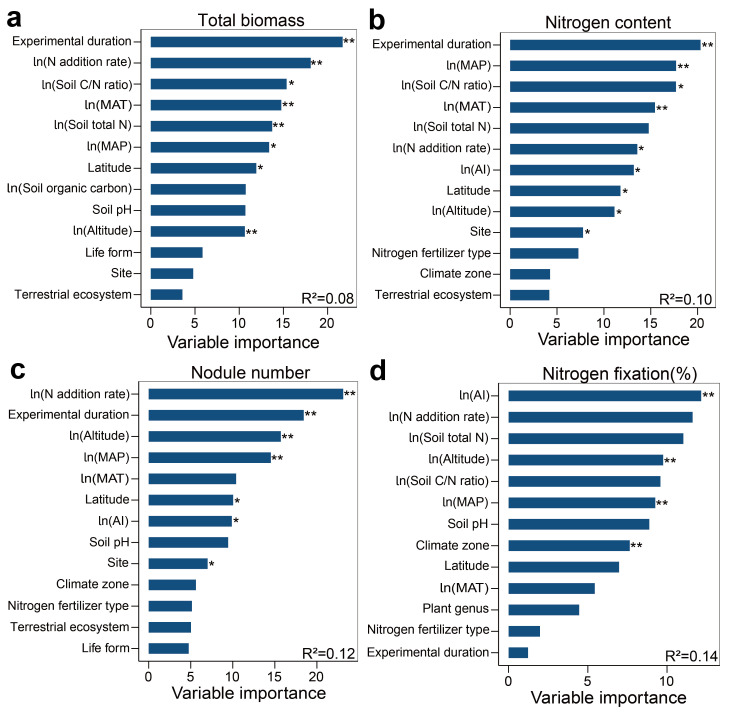
Differential importance of environmental factors and soil property response ratios for leguminous plants in response to nitrogen enrichment. * indicates *p* < 0.05, ** indicates *p* < 0.01. (**a**) Total biomass; (**b**) Nitrogen content; (**c**) Nodule number; (**d**) Nitrogen fixation (%).

**Figure 5:**
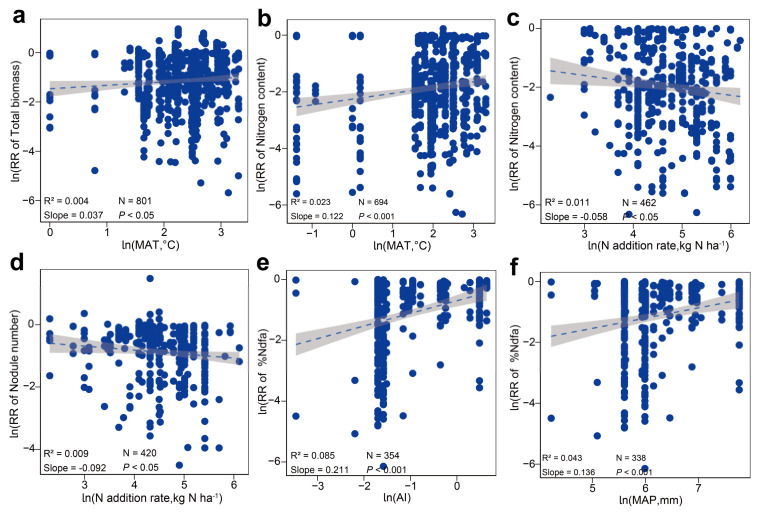
Relationship between growth traits, nitrogen fixation indicators and environmental factors in legumes under nitrogen enrichment. The dashed line and the grey thick-shaded line are the means of the slopes and the 95% confidence intervals of the correlation analyses, respectively. N indicates the sample size, slope > 0 indicates a positive correlation, slope < 0 indicates a negative correlation, and *p* < 0.05 indicates a significant correlation. (**a**) MAT and Total biomass; (**b**) MAT and Nitrogen content; (**c**) Nitrogen addition rate and Nitrogen content; (**d**) Nitrogen addition rate and Nodule number; (**e**) Al and %Ndfa; (**f**) MAP and %Ndfa.

Random forest analysis identified the MAT as a significant factor influencing the total biomass of legumes (Figure 4a). Correlation analysis revealed a significant positive relationship between the total biomass of legumes and the MAT (Figure 5a). The MAT is also a crucial factor influencing the N content in the tissues of leguminous plants (Figure 4b). Additionally, the rate of N fertilizer application affects tissue N content (Figure 5c). The N content in leguminous plant tissues significantly increases with rising MAT, while it markedly decreases with higher N fertilizer application rates (Figure 5b,c). Both the rate of N fertilizer application and altitude jointly drive the response of nodule numbers to N enrichment (Figure 4c). Correlation analysis reveals a significant negative correlation between nodule numbers and the rate of N fertilizer application (Figure 5d). AI and MAP are the primary drivers of the %Ndfa response to N enrichment in leguminous plants (Figure 4d), with %Ndfa showing a significant positive correlation with both AI and MAP (Figure 5e,f).

## 4. Discussion

### 4.1. N Enrichment on a Global Scale Promotes the Growth of Leguminous Plants but Diminishes Their N_2_-Fixing Capabilities

The responses of leguminous plants to N enrichment are complex and diverse. The findings of this study indicate that N enrichment significantly enhances the total biomass of leguminous plants and their tissue N content (Figure 2), which is consistent with numerous studies [89,90]. Although leguminous plants can meet their growth needs through N_2_ fixation in root nodules, they require high nutrient levels throughout their life cycle to sustain vigorous growth and achieve high yields [91,92]. Additional N supply can promote the growth of leguminous plants by alleviating N limitation, increasing biomass accumulation, and enhancing photosynthetic efficiency [93,94,95]. Furthermore, leguminous plants have a higher N demand compared to non-leguminous plants [96,97,98], as they require ample N resources to sustain elevated leaf N and photosynthetic capacity. Studies on natural tropical forests also indicate that the leaf N concentration of leguminous plants is often higher than that of non-leguminous plants [99,100,101], reflecting their greater N requirements. It is worth noting that leguminous plants not only have high N requirements but also possess a significant capacity to supply N [1], which positively affects soil and ecosystem health. For instance, the excess N released by rhizobia not only enhances soil fertility but also promotes the growth of non-leguminous plants [102]. Therefore, sufficient N fertilization is beneficial for the growth of leguminous plants [93,103]. Random forest analysis reveals that the MAT is a key factor influencing the response of total biomass and tissue N content of leguminous plants to N enrichment (Figure 4a,b). Correlation analysis shows that the total biomass and tissue N content of leguminous plants increase with a rising MAT (Figure 5a,b). One explanation for this is that temperature can directly affect the rate of photosynthesis in plants [104]. As temperatures rise, the rate of photosynthesis increases as it is a temperature-dependent physiological process. High temperatures can enhance the rate of enzyme-catalyzed reactions, thereby boosting the rate of photosynthesis and promoting plant growth and development [105]. Additionally, while elevated temperatures regulate plant growth, they also indirectly affect the mineralization of soil organic N [106,107]. Specifically, higher temperatures boost microbial metabolism and enzyme activity, accelerating the mineralization of soil organic N and increasing its availability. This, in turn, promotes plant growth and enhances N content in plant tissues. Our correlation analysis also shows a significant decrease in the N content of leguminous plant tissues with increasing N fertilizer application rates (Figure 5c). This may be due to excessive N input leading to N saturation [108], which hinders the ability of leguminous plants to absorb and utilize N efficiently. A study examining the physiological and ecological effects of N deposition on alpine meadow legumes suggests that short-term N addition enhances N absorption by legumes, whereas long-term N addition restricts their capability to absorb N [109]. Another explanation is that as biomass accumulates, the plants’ demand for nutrients rises, leading to increased N consumption. However, as biomass continues to increase, the concentration of N within the plant will gradually become reduced [110]. Furthermore, N content in the soil influences the allocation of biomass in plants [33]. Our findings demonstrate that N enrichment significantly reduces the root-to-shoot ratio of leguminous plants (Figure 2). When N levels in the environment are low, plants adapt by increasing the number of lateral roots and root hairs to access limited soil N, preferentially allocating photosynthetic products to the roots to enhance their N uptake capabilities [111]. However, when N availability in the environment increases, plants reduce their resource investment in roots and prioritize above-ground biomass, which aligns with predictions based on optimal allocation theory [33].

Our results also indicate that N addition significantly reduced nodule numbers and %Ndfa in leguminous plants (Figure 2 and Figure 5d), consistent with findings from numerous other studies [112,113]. The random forest model suggests that the rate of N addition is a key driver of the response of legume nodule numbers to N enrichment (Figure 4c). A common explanation for this phenomenon is that high N levels in the soil suppress oxygen diffusion within the root nodule cortex, ultimately leading to a decline in the respiration rate of root nodules and nitrogenase activity [114]. It is noteworthy that the nitrogenase activity in the root nodules of legumes refers to the ability of rhizobia to convert atmospheric N into plant-available N compounds within the nodules. This enzyme plays a crucial role in the N conversion process and is an essential component of the symbiotic N_2_-fixing system in legumes [115]. Additionally, this excess N inhibits the synthesis and accumulation of signal molecules, such as flavonoids, in leguminous plants. This interference thus restricts the recognition and infection processes between rhizobia and leguminous crops, resulting in a reduced number of nodules [116]. Similarly, N enrichment increases N availability in the soil, promoting some leguminous plants to adjust their N_2_-fixing strategies to utilize soil N for energy conservation, which consequently inhibits their N_2_-fixing capabilities [117]. Additionally, AI and MAP are significant factors influencing the %Ndfa response of legumes to N enrichment (Figure 4d). Correlation analysis shows a positive relationship between legumes %Ndfa and both AI and MAP (Figure 5e,f). On the one hand, drought conditions can inhibit plant photosynthesis. This is primarily because drought can cause the acidification of the chloroplast stroma, dehydration of the chloroplasts, and a reduction in their volume, all of which hinder photophosphorylation and disrupt the synthesis of adenosine triphosphate within the plant, thereby suppressing photosynthesis [118]. On the other hand, rhizobia relies on energy and carbon sources from the products of plant photosynthesis [119]. As a result, reduced photosynthesis can lead to an insufficient energy supply, which in turn affects N_2_ fixation in plants [120]. On the other hand, drought reduces soil moisture and hinders the movement of ions in the soil solution [121,122,123], which negatively affects the growth and activity of the root nodules, reducing the efficiency of their SNF. Additionally, N_2_-fixing bacteria are also susceptible to fluctuations in environmental humidity [124]. Specifically, nitrogenases are highly sensitive to oxygen, and their synthesis is inhibited in high-oxygen environments [125]. Conversely, increased precipitation creates a low-oxygen environment for N_2_-fixing bacteria [126], thus promoting N_2_ fixation efficiency. In summary, our findings support the first hypothesis: N enrichment promotes biomass increase in legumes while potentially reducing %Ndfa.

### 4.2. The Impact of N Enrichment on Legumes Exhibits Context Dependency

The results of this study demonstrate notable differences in the responses of total biomass to N enrichment across various ecosystem types. Legume biomass in temperate regions shows a significant response to N enrichment (Figure 3a), likely due to prevalent N limitation in these regions [127]. However, N enrichment shows no significant effect on the total biomass of legumes in tropical regions (Figure 3a). Previous research has largely attributed this phenomenon to the N saturation hypothesis. Tropical regions, in particular, tend to be richer in N compared to temperate regions, mainly due to abundant vegetation and biological N fixation. As a result, soils in tropical regions typically have higher N levels, and additional N inputs have little to no effect on the growth of leguminous plants [6,128]. Additionally, our findings highlight that climatic factors play a crucial role in influencing the responses of legume biomass to N enrichment. Specifically, a higher MAT and MAP are associated with greater increases in total biomass of legumes (Figure 3a). The correlation analysis also confirmed that the total biomass of legumes significantly increases with a rising MAT (Figure 5a). Higher temperatures promote elevated plant photosynthetic rates [104], directly enhancing their growth and development. At the same time, the MAT regulates soil N mineralization, with an increased MAT leading to higher levels of available N for plant uptake [129]. In contrast, low temperatures can reduce the activity of photosynthesis-related enzymes, lower chlorophyll content, and decrease stomatal conductance [130], ultimately diminishing photosynthetic rates and negatively affecting plant yield. Low temperatures may also restrict the secretion of flavonoids in leguminous plants and the survival of N_2_-fixing rhizobia, hindering the exchange of specific signaling molecules between them, ultimately leading to a reduction in nodule numbers and adversely affecting the growth of leguminous plants [131,132]. Additionally, low temperatures have been shown to significantly suppress the rate of N absorption and assimilation in plants [133,134]. Wu and colleagues found that the ratio of NH_4_^+^ absorbed by plants exceeds that of NO_3_^−^ at various temperatures, and this trend becomes more pronounced as temperatures decrease [135]. In contrast, other researchers have discovered that lowered temperatures inhibit the uptake of NH_4_^+^ through high-affinity transport systems, resulting in a marked decrease in NH_4_^+^ absorption while increasing the affinity for NO_3_^−^ [136,137]. Thus, it is clear that low temperatures also affect plant responses to different forms of N. The increase in MAP provides essential moisture for plant photosynthesis, thereby promoting their growth and development [71]. It can also accelerate root metabolism and enhance soil organic carbon content [74,138]. Consequently, higher MAP may improve soil moisture and nutrient availability, leading to an increase in the total biomass of leguminous plants (Figure 3a). Notably, in our study, N enrichment led to a significant increase in the total biomass of leguminous plants in semi-arid regions (Figure 3a), consistent with the findings of Li et al. [129]. One explanation is that N supply can enhance the drought resistance of leguminous plants [139,140]. Specifically, appropriate N supplementation improves the ratio of N allocated to Rubisco in the leaves (i.e., enhancing Rubisco’s carboxylation capacity), thereby enhancing photosynthetic C assimilation and increasing plant tolerance to drought stress [141,142]. Another study also showed that even under drought stress, *Cajanus cajan* can maintain good yields [143], providing further evidence to support our research. In summary, our findings partially support the second hypothesis: in regions with abundant water and favorable thermal conditions, N enrichment promotes an increase in the biomass of leguminous plants.

Our results also showed that there are variations in the responses of different leguminous plant species to N enrichment. Specifically, woody legumes exhibit a greater total biomass response to N enrichment compared to herbaceous legumes (Figure 3a), which is associated with their higher N preference [144,145]. Additionally, the response of legume biomass to N enrichment is substantially enhanced within cropland ecosystems (Figure 3b). Research has shown that the application of N fertilizer in cropland ecosystems not only improves soil conditions [146] but also increases the abundance of varying resources in the soil food web, thereby facilitating plant growth and development [147,148]. Moreover, in cropland ecosystems, legumes can mitigate the negative impacts of N fertilizer (e.g., N depression) through intercropping with other crops [149]. In contrast, legumes in natural grassland ecosystems generally show insensitivity to N enrichment (Figure 3b). This may be due to additional resource limitations (e.g., water, temperature, nutrients) and the competitive constraints imposed by competing herbaceous plants such as superior graminoid competitors [150,151]. Interestingly, under N enrichment conditions, the total biomass of legumes in acidic soils is not inhibited (Figure 3b). Stevens et al. [152] have identified soil pH as a key factor driving plant productivity. On one hand, regions with acidic soils often have abundant resources such as water, light, and heat [153]. Our subgroup analysis has confirmed that legume biomass significantly increases in these resource-rich environments (Figure 3a). On the other hand, this phenomenon is associated with the physiological mechanisms that enable legumes to adapt to acidic soils, such as improving N utilization efficiency in these environments [154]. As the N application rate and the duration of the experiment increase, the total biomass of leguminous plants is suppressed (Figure 3b). This is probably because prolonged high N input can lead to excess soil N concentrations that hinder plant growth and development, ultimately reducing the net photosynthetic rate and biomass [27]. Furthermore, the form of N significantly affects plant growth and adaptation (Figure 3b) [155]. The application of urea and mixed N forms facilitates the dissolution of certain organic matter in the soil [156,157], thereby enhancing the availability of nutrients for plant uptake. Notably, leguminous plants exhibit the greatest response to NH_4_^+^ (Figure 3b), which correlates with the lower respiratory cost associated with NH_4_^+^ absorption compared to NO_3_^−^. Specifically, NO_3_^−^ must first be reduced to NH_4_^+^ via nitrate reductase (NR) and nitrite reductase (NiR) before it can undergo assimilation to become an available organic form of N for plants [158]. Compared to N sources that require more energy for assimilation, plants preferentially choose the least costly options to optimize growth and competitive survival, thereby maintaining a competitive advantage [159,160]. Additionally, the sole application of NH_4_^+^ compared to NO_3_^−^ results in higher nodule weight, total N accumulation, and N_2_ fixation in *soybean* [161,162]. The site of the experiment also influences biomass response to N enrichment. Our study showed that in contrast to in situ experiments, ex situ N supply substantially enhances biomass accumulation (Figure 3b). This is likely because ex-situ conditions mitigate external environmental factors, such as rainwater runoff, which reduces N loss and thereby amplifies the effect of N supply [163]. Moreover, legumes exhibit a higher leaf N content with in situ N addition compared to non-leguminous plants, making them more attractive to herbivorous animals for foraging [101,164], which could lead to biomass loss for legumes. In summary, due to variations in environmental conditions, N application practices, and the differing N demands and preferences of leguminous plants, their response to exogenous N inputs is context-dependent.

### 4.3. Research Prospects

In most agricultural ecosystems, N is a key element for crop growth and development. However, excessive application of N fertilizers can reduce N use efficiency in crops, contribute to soil acidification, loss of biodiversity, and exacerbate climate change [165,166]. Furthermore, N fertilizers are prone to losses through various pathways, including NH_4_^+^ volatilization, NO_3_^−^ leaching, denitrification, and water runoff [167,168,169]. These processes contribute to N pollution in cropland ecosystems, which remains a pressing issue. Therefore, the application of N fertilizers in crop production must take into account their environmental impacts, which could also help mitigate climate change. It is well known that the N_2_-fixing ability of legumes is regarded as a relatively eco-friendly N source in agricultural production [170]. This is primarily due to legumes’ ability to reduce dependence on inorganic N through N_2_ fixation, thereby decreasing the need for chemical fertilizers in agricultural practices [3]. Additionally, intercropping legumes with cereal crops can substantially reduce N fertilizer use while maintaining or even boosting crop yields compared to monoculture systems [171]. We propose implementing crop rotation by alternating legumes with other crops in agricultural practices. This approach would fully leverage the N_2_-fixing capability of legumes, reduce N fertilizer application, optimize N supply for subsequent crops, and ultimately alleviate N pollution in agriculture. Consequently, addressing the scientific question regarding the growth traits of legumes and their response to N accumulation can provide valuable insights and theoretical foundations for N enrichment management in agricultural systems amid global climate change, thereby facilitating sustainable agricultural development. There are several limitations to our study. First, the number of experimental observations may affect the strength of correlations between leguminous plants and other climatic/environmental factors. Therefore, the conclusions should be interpreted with caution. Second, while a meta-analysis based on the most comprehensive dataset was conducted to assess the impact of N enrichment on leguminous plant growth, the distribution map of research sites reveals a lack of data from regions such as Russia and northern Canada. Therefore, we strongly recommend increasing field observations in these underrepresented regions to improve our understanding of how nitrogen enrichment affects the growth and development of leguminous plants. Lastly, while our study focuses on the effects of N enrichment, temperature, precipitation, and drought on the growth and N_2_-fixing capacity of leguminous plants, other factors, such as light intensity [172] and carbon dioxide concentration [173], may also play important roles, which warrants further investigation.

## 5. Conclusions

Our meta-analysis quantifies the impact of N enrichments on legume biomass and their N_2_-fixing capacity while elucidating the responses of legume biomass across diverse ecosystems. The findings reveal that the initial effect of N enrichments on the total biomass of legumes is positive. However, this beneficial impact tends to diminish as the rate of N addition and the duration of the experiment increase. Concurrently, N enrichments negatively affect the N_2_-fixing capacity of legumes, evidenced by a reduction in root-to-shoot ratio, nodule number, nodule weight, and %Ndfa. Moreover, the influence of N enrichments on legumes is regulated by factors such as the rate of N fertilizer application, climatic conditions, AI, and soil physicochemical properties, resulting in variability in the response of legume biomass to N accumulation influenced by factors including region, plant species, climatic conditions, ecosystem types, soil pH, and experimental conditions. In light of future pressures from global climate change, intensified N enrichment, and worsening drought conditions, leguminous plants, which play a crucial role in SNF, will face greater challenges. This would subsequently impact the stability and sustainability of agricultural production.

## Data Availability

Dataset available on request from the authors.

## Supplementary Materials

The following supporting information can be downloaded at https://www.mdpi.com/article/10.3390/plants13223244/s1, Figure S1 Preferred reporting items for systematic reviews and meta-analyses (PRISM) diagram showing the process and locating in this meta-analysis; Figure S2 Interpolation results of soil nutrients and physicochemical properties obtained by chain equation multivariate interpolation method based on random forest; Figure S3 Overall analysis preference tests. Horizontal coordinates indicate standard errors, and vertical coordinates indicate response ratios. The top of each panel contains the results of publication bias tests using Egger’s regression (*z* and *p*-value). *p* < 0.05 indicates the presence of publication bias. Percentage of Nitrogen Fixation (%Ndfa); Figure S4 Subgroup analysis preference tests of total biomass. Horizontal coordinates indicate standard errors, and vertical coordinates indicate response ratios. The top of each panel contains the results of publication bias tests using Egger’s regression (*z* and *p*-value). *p* < 0.05 indicate the presence of publication bias; Figure S5 Effect sizes and confidence intervals for overall analyses of the effects of nitrogen enrichment on the growth and nitrogen fixation capacity of legumes; Figure S6 Effect sizes and confidence intervals for subgroup analyses of the effect of nitrogen enrichment on total biomass in legumes.
