# Supplementary material for: Patterns and Mechanisms of Legume Responses to Nitrogen Enrichment: A Global Meta-Analysis
Source: Plants (Basel). 2024 Nov 19;13(22):3244. doi: 10.3390/plants13223244 (PMC11598177; doi:10.3390/plants13223244)
Supplement: Supplementary file 1 [file plants-13-03244-s001.zip › plants-3256058-supplementary.pdf]

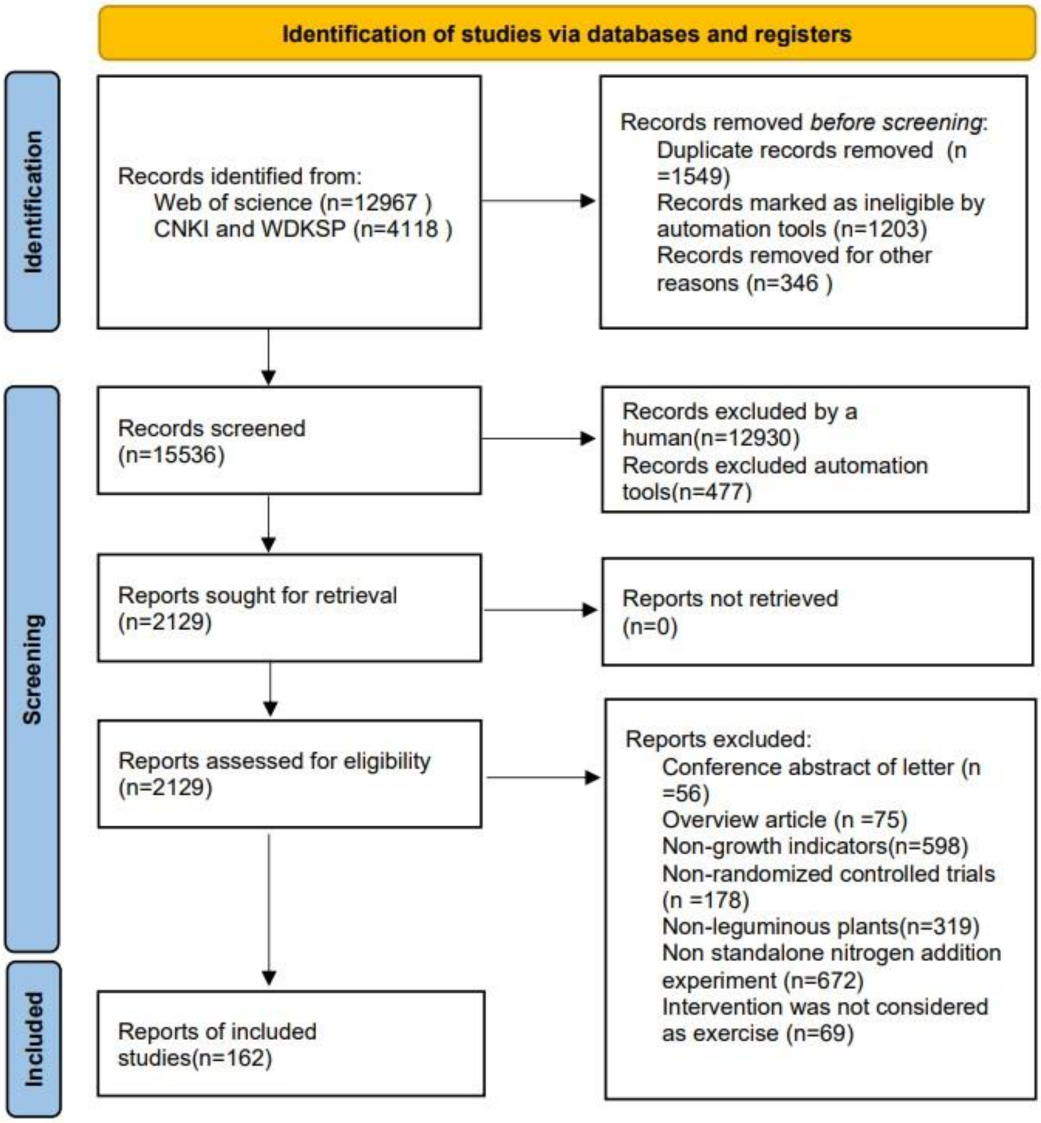

Fig. S1 Preferred reporting items for systematic reviews and meta-analyses (PRISM) diagram showing the process and locating in this meta-analysis.

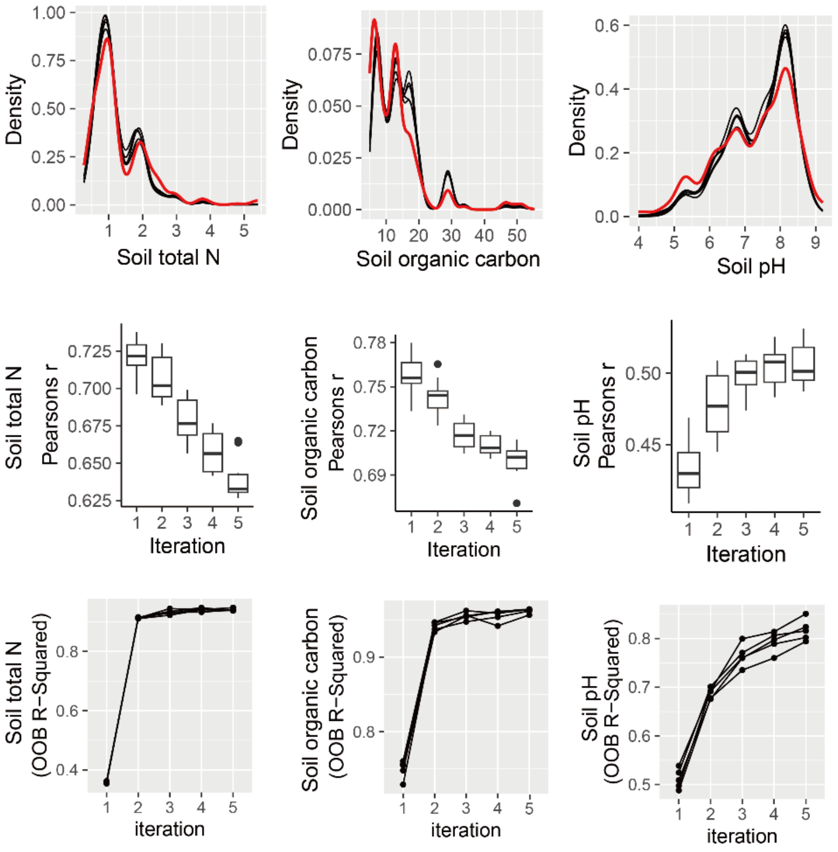

Fig. S2 Interpolation results of soil nutrients and physicochemical properties obtained by chain equation multivariate interpolation method based on random forest.

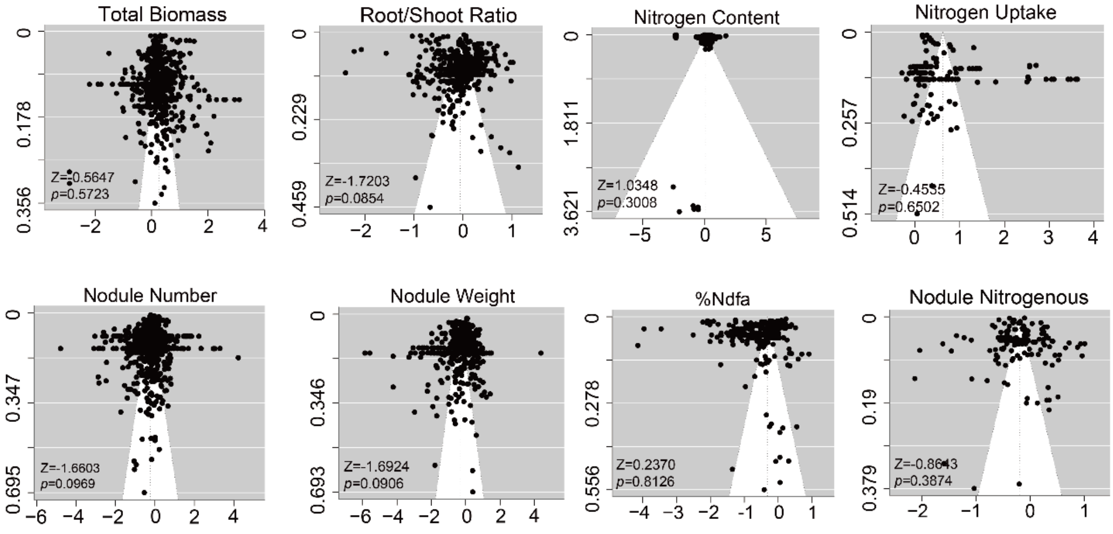

Fig. S3 Overall analysis preference tests. Horizontal coordinates indicate standard errors and vertical coordinates indicate response ratios. The top of each panel contains the results of publication bias tests using Egger's regression (*z* and *p*-value). $p<0.05$

indicate the presence of publication bias. Percentage of Nitrogen Fixation (%Ndfa).

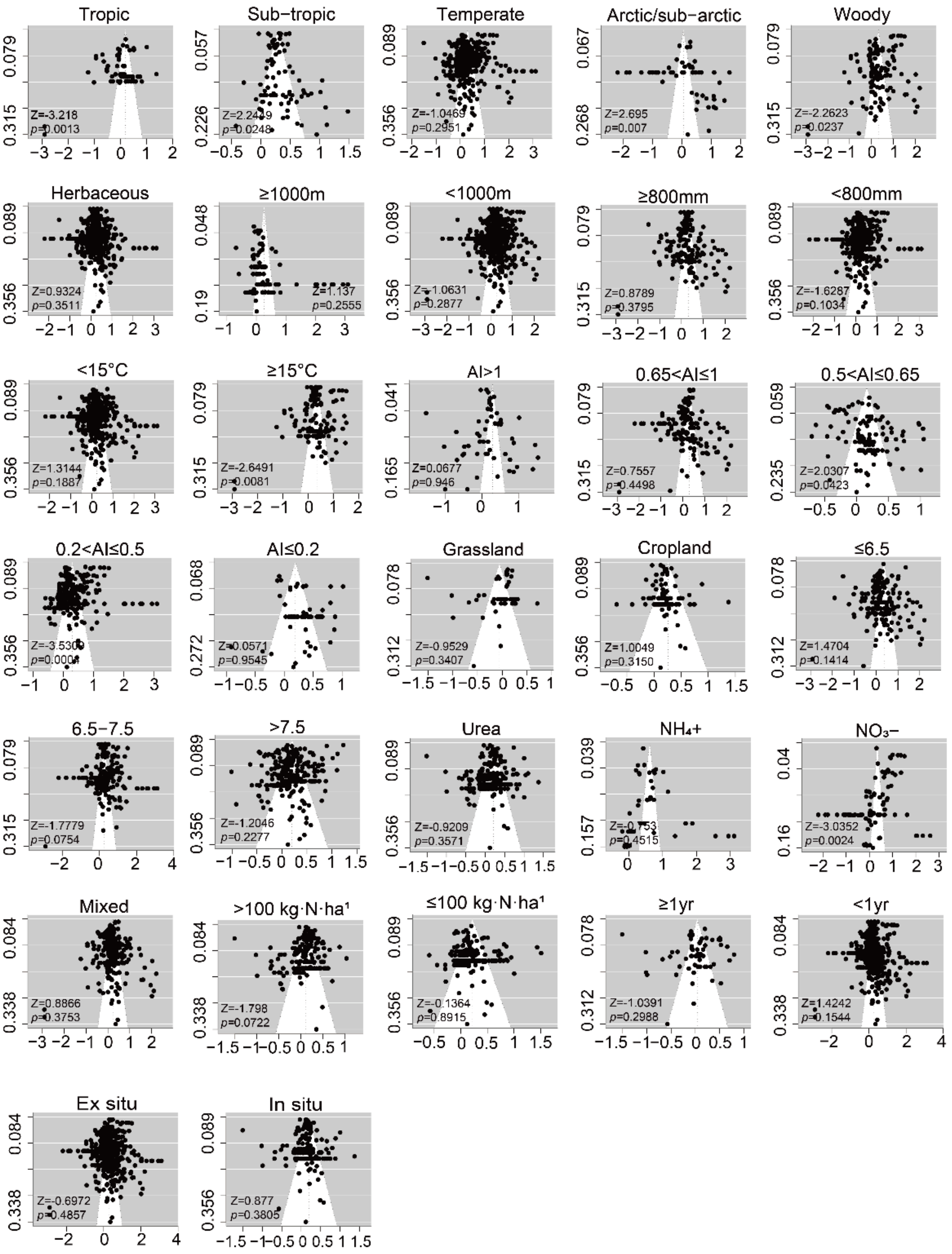

Fig. S4 Subgroup analysis preference tests of total biomass. Horizontal coordinates indicate standard errors and vertical coordinates indicate response ratios. The top of each panel contains the results of publication bias tests using Egger's regression (*z* and *p*-value). $p<0.05$ indicate the presence of publication bias.

Indicators	Effect sizes	95% confidence interval
Total biomass	0.2694	0.1706~0.3682
Root/shoot ratio	-0.0601	-0.1133~-0.0068
Nitrogen content	0.1242	0.0144~0.234
Nitrogen uptake	0.6204	0.1983~1.0424
Nodule number	-0.2376	-0.3842~-0.0911
Nodule weight	-0.3469	-0.5199~-0.174
Nitrogen fixation (%)	-0.3156	-0.4602~-0.1711
Nodule nitrogenous	-0.1888	-0.4298~0.0521

Fig. S5 Effect sizes and confidence intervals for overall analyses of the effects of nitrogen enrichment on the growth and nitrogen fixation capacity of legumes.

Indicators	Indicator types	Effect sizes	95% confidence interval
Latitude	Tropic	0.1879	-0.1149~0.4907
	Sub-tropic	0.2852	0.1547~0.4157
	Temperate	0.3044	0.1725~0.4363
	Arctic/sub-arctic	0.0495	-0.3630~0.4619
Plant species	Woody	0.3197	0.0961~0.5433
	Herbaceous	0.2300	0.1302~0.3297
Altitude	≥ 1000m	0.2594	-0.1047~0.6236
	< 1000m	0.2710	0.1737~0.3684
MAP	≥ 800mm	0.3125	0.1223~0.5027
	< 800mm	0.2502	0.1434~0.3569
MAT	< 15°C	0.2402	0.1227~0.3577
	≥ 15°C	0.3499	0.1736~0.5261
AI	AI > 1.0	0.2838	-0.0464~0.6141
	0.65 < AI ≤ 1.0	0.3036	0.1032~0.5041
	0.50 < AI ≤ 0.65	0.1586	0.0118~0.3055
	0.20 < AI ≤ 0.50	0.2976	0.1314~0.4637
	AI ≤ 0.2	0.1918	-0.0041~0.3877
Terrestrial ecosystem	Grassland	-0.0569	-0.3305~0.2167
	Cropland	0.2609	0.0945~0.4273
Soil pH	≤ 6.5	0.3670	0.1782~0.5558
	6.5-7.5	0.2576	0.0759~0.4394
	> 7.5	0.2004	0.1170~0.2838
Nitrogen fertilizer type	Urea	0.2097	0.0915~0.3279
	NH_4^+-N	0.6152	0.1481~1.0822
	NO_3^--N	0.3481	0.0139~0.6823
	Mixed	0.2264	0.0734~0.3793
Nitrogen addition rate	> 100 kg N ha^{-1}	0.1171	0.0111~0.2231
	≤ 100 kg N ha^{-1}	0.2199	0.1397~0.3002
Experimental duration	≥ 1year	0.0402	-0.1451~0.2256
	< 1year	0.2775	0.1580~0.3971
Experimental site	Indoor	0.3028	0.1752~0.4305
	Field	0.1956	0.0640~0.3272

Fig. S6 Effect sizes and confidence intervals for subgroup analyses of the effect of

nitrogen enrichment on total biomass in legumes.
